# Biologic Augmentation in Anterior Cruciate Ligament Reconstruction and Beyond: A Review of PRP and BMAC

**DOI:** 10.3390/jcm14196959

**Published:** 2025-10-01

**Authors:** Grant M. Pham

**Affiliations:** Regenerative Medicine, Arlington Regenerative Medicine Institute, Arlington, TX 76013, USA; grantmpham@gmail.com

**Keywords:** anterior cruciate ligament reconstruction, platelet-rich plasma, platelet-rich fibrin, bone marrow aspirate concentrate, biologic augmentation, regenerative sports medicine

## Abstract

This narrative review synthesizes PubMed- and Scopus-indexed studies from 2020 to 2025, including preclinical animal models, prospective cohort studies, and level I and II randomized trials, to compare two leading biologic augmentation strategies: platelet-rich plasma (PRP) and bone marrow aspirate concentrate (BMAC). The review examines underlying mechanisms of action, delivery techniques, imaging biomarkers of graft maturation, patient-reported and functional outcomes, safety profiles, cost-effectiveness, and regulatory frameworks. PRP provides early anti-inflammatory and proangiogenic signaling, while BMAC delivers a concentrated population of mesenchymal stem cells and growth factors to the tendon–bone interface. Both modalities consistently enhance MRI-defined graft maturation, yet evidence of long-term functional or biomechanical superiority remains inconclusive. Emerging therapies such as peptide hydrogels, adipose-derived stem cells, and exosome delivery offer promising avenues for future research. Standardized protocols and large multicenter trials are needed to clarify comparative efficacy and inform personalized rehabilitation strategies.

## 1. Introduction

### Introduction and Background

Anterior cruciate ligament (ACL) injuries represent a major orthopedic and public health challenge, particularly among athletes. Annually, over 200,000 ACL injuries are diagnosed in the United States, resulting in a significant number of surgical reconstructions, many of which occur in individuals aged 15 to 35 [1,2]. The ramifications of these injuries extend beyond mechanical instability, often affecting athletic careers, daily mobility, and long-term joint health. Anterior cruciate ligament injury is also a known risk factor for post-traumatic osteoarthritis, especially when combined with meniscal or cartilage damage [3]. Thus, successful ACL reconstruction is essential for restoring joint kinematics and preventing degenerative changes.

Over the past two decades, surgical advancements such as anatomical tunnel placement using anteromedial and all-inside approaches, improved graft fixation technologies including cortical suspensory systems and interference screws, and the adoption of minimally invasive arthroscopic techniques have substantially enhanced the precision and reproducibility of ACL reconstruction [4]. However, achieving consistent biological integration of the graft remains a clinical challenge [2]. Issues at the tendon-to-bone interface, including inadequate fibrocartilage formation, excessive inflammation, and graft motion, continue to hinder the formation of a robust, native-like enthesis [5]. The restoration of the native enthesis, or tendon-to-bone interface, is vital for long-term graft survival. Healing at this interface is slow and often incomplete, limiting early functional recovery and increasing re-rupture rates in young, active populations.

The biologic remodeling of the tendon graft after ACL reconstruction proceeds through distinct stages, initial necrosis and inflammation, followed by vascular invasion, cellular repopulation, and extracellular matrix reorganization, culminating in graft maturation or ligamentization. Despite this orchestrated sequence, the process often remains incomplete, and the healed graft rarely matches the structural and mechanical profile of the native ACL [5,6,7]. Factors such as patient age, comorbidities, smoking status, and timing of surgery can further complicate biologic healing. Therefore, strategies that accelerate and improve graft incorporation have become a focus of modern regenerative and orthopedic medicine.

Biologic augmentation aims to enhance the local healing environment using autologous or engineered biologics to support graft integration. Platelet-rich plasma (PRP) and bone marrow aspirate concentrate (BMAC) are among the most studied in this field [8]. Platelet-rich plasma offers a concentrated source of growth factors, including vascular endothelial growth factor (VEGF) and transforming growth factor beta 1 (TGF-β1), that stimulate angiogenesis and tissue repair [9]. Bone marrow aspirate concentrate provides a heterogeneous mix of cells, including mesenchymal stem cells (MSCs), capable of differentiating into ligamentous tissue and modulating inflammation. Emerging preclinical evidence suggests that these biologics may reduce tunnel widening, promote fibrocartilage formation, and increase mechanical strength at the graft interface [10].

Foundational reviews have consolidated the mechanistic rationale for biologic therapies. Andriolo et al. (2020) and Dulic et al. (2025) underscored the potential for PRP and MSCs to influence healing outcomes, while Rodríguez-Merchán (2021) critically evaluated clinical evidence and called for standardized methodologies [11,12,13]. In parallel, novel delivery platforms, including bioresorbable scaffolds, growth factor-binding peptides, and hydrogel matrices, have been developed to prolong the residence time of biologics and optimize their spatial distribution at the tendon–bone junction.

Recent interest has also turned to combining biologics with rehabilitation protocols to synchronize mechanical loading with peak biologic activity. For example, early neuromuscular stimulation may enhance cell migration and improve matrix alignment in biologically augmented grafts. Imaging tools such as quantitative MRI and ultrasound elastography are also being explored to monitor biologic activity and guide return-to-sport decisions. Collectively, these approaches reflect a shift toward personalized ACL reconstruction that integrates surgical, biologic, and rehabilitative strategies. Therefore, the aim of this review is to synthesize current evidence on biologic augmentation strategies for ACL reconstruction, with a specific focus on their role in enhancing graft healing and integration at the critical tendon–bone interface. It will evaluate the mechanistic rationale, preclinical findings, and clinical outcomes of prominent biologics such as PRP and BMAC. Furthermore, this review will explore emerging trends, including novel delivery systems and the integration of biologic therapies with personalized rehabilitation protocols, to provide a comprehensive overview of the present and future of biologics in optimizing ACL reconstruction outcomes.

## 2. Methods

This review synthesizes data from 2020 to 2025 drawn from studies indexed in PubMed and Scopus, including preclinical animal models, prospective cohort studies, and randomized controlled trials at levels I and II, to compare platelet-rich plasma (PRP) and bone marrow aspirate concentrate (BMAC) in ACL reconstruction. A search of both databases identified records from January 2020 through July 2025; titles and abstracts were screened, and full texts were reviewed by the author. The exact combinations used were (anterior cruciate ligament OR ACL) AND (reconstruct* OR repair*) AND (platelet-rich plasma OR PRP OR platelet-rich fibrin OR PRF); (anterior cruciate ligament OR ACL) AND (reconstruct* OR repair*) AND (bone marrow aspirate concentrate OR BMAC OR bone marrow aspirate); (anterior cruciate ligament OR ACL) AND (reconstruct* OR repair*) AND (mesenchymal stem cell* OR mesenchymal stromal cell* OR MSC); and (anterior cruciate ligament OR ACL) AND (reconstruct* OR repair*) AND (biologic augmentation OR biological augmentation). The review explicitly lists preclinical studies, randomized trials, prospective and retrospective cohorts, meta-analyses, and prior reviews, with priority given to level I and II clinical evidence where available. It then focuses on mechanisms of action, delivery strategies, clinical outcomes, imaging markers, and synchronization with rehabilitation protocols, and evaluates safety, cost-effectiveness, and regulatory considerations while highlighting key gaps and future directions in regenerative sports medicine.

## 3. Discussion

### 3.1. Biologic Mechanisms of PRP and BMAC

Platelet-rich plasma (PRP) and its scaffold-forming derivative platelet-rich fibrin (PRF) are prepared from autologous whole blood using centrifugation protocols that isolate the platelet-rich component [14]. These platelets harbor a reservoir of bioactive molecules, including platelet-derived growth factor (PDGF), TGF-β, VEGF, insulin-like growth factor 1 (IGF-1), epidermal growth factor (EGF), and fibroblast growth factor (FGF), each of which plays a role in modulating inflammation, angiogenesis, and extracellular matrix (ECM) synthesis [15]. Upon activation, either through calcium chloride or mechanical stress, these platelets release their growth factors, initiating a cascade that promotes cellular chemotaxis and matrix deposition.

In ACL reconstruction, PRP can be applied by soaking grafts, injecting into bone tunnels, or layering around the graft–tunnel interface [16]. Platelet-rich fibrin forms a three-dimensional fibrin network that functions as a slow-release system, prolonging growth factor bioavailability and acting as a scaffold for progenitor cells [17]. The resulting biologic environment is enriched for regenerative signaling, though the magnitude and duration of these effects depend heavily on PRP formulation, particularly whether leukocytes are included. Leukocyte-rich PRP contains higher levels of white blood cells and pro-inflammatory cytokines, while leukocyte-poor PRP has minimal leukocyte content and promotes anti-inflammatory and anabolic responses. Other critical variables include platelet concentration and timing of delivery. Numerous analyses since 2020 have demonstrated that leukocyte-poor PRP yields less inflammation, reduces catabolic cytokine release, and better supports tissue healing than leukocyte-rich PRP [18].

Preclinical animal models support these findings. In rabbit ACL reconstruction models, PRP and PRF-treated grafts exhibited higher vascular density and collagen fiber organization at postoperative follow-up [19,20]. Canine studies have shown improved pull-out strength and histologic evidence of early fibrocartilage formation in PRP-augmented tunnels [21]. However, some studies have reported minimal differences in mechanical properties at longer follow-ups, highlighting the need for sustained biologic stimuli or combination therapies [22].

Bone marrow aspirate concentrate (BMAC) contains MSCs, endothelial progenitor cells, mononuclear cells, and a broad spectrum of cytokines [23]. Unlike PRP, BMAC provides both a cellular and humoral component, potentially offering a more sustained regenerative effect [10]. MSCs contribute to healing by differentiating into ligamentous fibroblasts and by secreting exosomes and paracrine factors that reduce inflammation and encourage ECM remodeling. Several mechanistic pathways have been proposed to explain BMAC’s effects [23]. Stromal cell-derived factor 1 (SDF-1), secreted by local tissues, may attract circulating MSCs to the graft site, where they participate in tissue remodeling. Growth factors like basic fibroblast growth factor (bFGF) and hepatocyte growth factor (HGF) enhance angiogenesis and promote fibroblast proliferation. In vivo, BMAC-treated ACL grafts demonstrate increased mineralized tissue at the bone–tendon junction, reduced tunnel widening, and improved biomechanical properties. Optimization of BMAC preparation is critical. Studies have shown that factors such as volume of aspirate, centrifugation speed, and anticoagulant use influence cell viability and growth factor concentration. Moreover, combining BMAC with bioresorbable scaffolds or hydrogels has been shown to improve cell retention and distribution, enhancing its efficacy [23]. Newer approaches involve pre-conditioning BMAC with hypoxic environments or mechanical stimulation to prime cells before implantation.

### 3.2. Clinical Applications & Delivery Techniques

Clinically, PRP and PRF are widely utilized during ACL reconstruction for their regenerative potential [24]. Platelet-rich plasma is typically administered intraoperatively by soaking graft materials or injecting directly into femoral and tibial bone tunnels, with the aim of enhancing the biologic environment and supporting early-phase healing. Platelet-rich fibrin, by contrast, is prepared as a gel or membrane that can be applied around grafts or within tunnels, offering a dual role as both a biologic reservoir and a structural scaffold [25]. A randomized study by Anz et al. (2025) showed preoperative PRP injections in patients with acute ACL tears significantly reduced synovial inflammation and chondrodegenerative biomarkers, suggesting a protective intra-articular effect prior to reconstruction [9]. Postoperatively, adjunctive intra-articular injections of PRP are sometimes used at regular one-month intervals to sustain the healing cascade, particularly in the early phases of ligamentization [22,24]. Such practices are more common in high-performance athletes or in patients with slower initial recovery [14].

The methods used to prepare PRP and PRF, such as the choice of centrifugation speed, platelet concentration targets, and inclusion or exclusion of leukocytes, significantly impact the final product’s composition and bioactivity [25]. Leukocyte-rich PRP may provoke a stronger inflammatory response, which could be beneficial or detrimental depending on timing and delivery site. Double-spin centrifugation typically produces higher concentrations of platelets but may reduce growth factor diversity (Table 1). Platelet-rich fibrin, with its fibrin mesh, offers slow-release kinetics and provides a more durable carrier for cellular and molecular components [17,25].

Bone marrow aspirate concentrate (BMAC) is harvested intraoperatively, most often from the posterior or anterior iliac crest, and concentrated using centrifugation to isolate a nucleated cell-rich fraction. It is typically injected into the bone tunnels or applied directly to graft surfaces before fixation. In some advanced protocols, BMAC is incorporated with scaffolds such as collagen membranes or demineralized bone matrix to enhance retention and prolong cellular viability. The delivery strategy of BMAC often depends on the surgical technique and graft type. For example, in all-inside techniques using suspensory fixation, BMAC may be injected retrograde into the tunnel following graft insertion. In full tibial tunnel approaches, it can be combined with bone chips or soaked into the graft construct prior to passage. Emerging technologies like intraosseous injection systems and scaffold-assisted delivery platforms aim to improve spatial localization and integration of biologics [26]. Moreover, in select cases of partial ACL tears or chronic laxity where surgery is deferred, ultrasound-guided BMAC or PRP injections have been used as part of non-operative biologic management strategies. Early reports suggest these approaches may offer symptom relief and imaging-based evidence of healing, though randomized controlled trials are needed to substantiate efficacy [27].

Standardization of biologic delivery remains a key challenge. Variability in concentration, preparation technique, timing of application, and host factors all influence treatment response. Future directions may include point-of-care cell characterization, real-time biologic activity assays, and integration with personalized rehabilitation timelines to tailor delivery for maximal impact.

Taken together, PRP and BMAC represent complementary strategies for biologic augmentation. PRP acts quickly, providing a burst of growth factors during the early healing phase, while BMAC offers a more durable cellular response. PRP can transiently increase PDGF, TGF-β, VEGF, and downshift inflammatory markers [9]. Understanding how these agents influence the molecular and mechanical milieu of ACL grafts will be essential for developing next-generation biologic therapies, as depicted in Table 1. Further studies are needed to compare their efficacy directly, identify optimal dosing and delivery schedules, and assess their synergy with evolving rehabilitation paradigms.

### 3.3. Clinical Evidence and Comparative Outcomes

A meta-analysis by Lv et al. (2021) of 17 randomized controlled trials including 970 ACL reconstruction patients found that PRP significantly reduced early postoperative pain (mean VAS decrease of ~1.1 points) and improved short to mid-term IKDC and Lysholm scores, though long-term functional significance remained limited [28]. These findings highlight the potential value of PRP in facilitating early recovery and symptomatic relief. However, these benefits were not sustained at 12 months, and PRP did not improve tunnel widening or objective knee stability. Functional outcomes, such as return-to-sport readiness and re-injury rates, showed no significant difference compared to control groups in long-term follow-up. These results suggest that while PRP may support early-phase biological remodeling, its utility in driving long-term clinical success remains limited (Table 1). Additionally, the heterogeneity in PRP formulations, preparation techniques, and application timing further complicates the interpretation of existing data [29,30]. Compounding this, Kasl et al. (2022) evaluated Vivostat^TM^, a platelet-rich fibrin prepared intraoperatively from autologous venous blood in a closed system (centrifugation and controlled polymerization yielding a sprayable fibrin matrix) [31]. The PRF was applied to the graft and tunnel apertures during ACLR. They found no significant differences in MRI ligamentization or patient-reported outcomes at 6 and 12 months versus controls, and recommended larger trials focused on the immediate postoperative window, where short-lived effects might be detected. In contrast, Milovanovic et al. (2024) used the same Vivostat PRF technique during bone patellar tendon–bone ACL reconstruction, with additional application to the patellar and tibial donor sites [32]. They reported reduced kneeling pain and faster narrowing of harvest site bone defects at 8 and 12 months, but no statistically significant differences immediately after surgery or at 4 months. Together, these studies suggest any PRF benefit may be time dependent and more evident in later structural remodeling than in early postoperative imaging or symptoms.

Bone marrow aspirate concentrate (BMAC) has shown promise in enhancing biological graft incorporation. Several small-scale trials and prospective studies have reported improved MRI-based parameters, including lower signal intensity and increased synovial coverage of the graft [33]. Enhanced bone tunnel integration and reduced tunnel widening were also noted in patients receiving BMAC augmentation. One particular study by Jordan et al. (2025) involving the application of BMAC with a collagen matrix in allograft ACL reconstruction demonstrated appeared safe and yielded favorable clinical outcomes at two-year follow-up [23]. Moreover, there is some emerging evidence that BMAC may influence the timeline for graft maturation, potentially enabling earlier initiation of dynamic rehabilitation [33]. Nonetheless, the magnitude of clinical improvement remains modest, and studies vary significantly in methodological quality.

Importantly, direct head-to-head comparisons between PRP and BMAC remain rare [29]. Existing trials often differ in inclusion criteria, graft type, outcome measures, and follow-up duration, making it difficult to draw definitive conclusions regarding superiority. Only a few comparative analyses have attempted to evaluate PRP versus BMAC within the same clinical protocol. These studies tend to suggest that BMAC may confer greater benefits in terms of imaging and tunnel morphology, whereas PRP provides more pronounced short-term symptomatic improvement [30]. There is currently insufficient evidence to determine whether one biologic is clearly superior across all outcome domains.

Moving forward, more robust randomized controlled trials are needed to clarify the comparative efficacy of these interventions. Ideally, future studies should employ standardized biologic preparation methods, consistent imaging modalities, and validated functional scoring systems. In addition, the integration of biomechanical testing, molecular biomarkers, and long-term outcome tracking will be critical to fully elucidate the therapeutic value of biologic augmentation in ACL reconstruction. Until such data are available, clinicians must continue to individualize biologic strategies based on patient-specific factors, graft selection, and surgical technique.

### 3.4. Safety, Cost, and Regulatory Issues

Platelet-rich plasma (PRP) enjoys a favorable safety profile and is generally well-tolerated by patients (Table 1). Minor adverse effects reported include localized swelling, mild discomfort at the injection site, or transient joint pain, all of which are typically self-limiting [34]. Due to its autologous nature, the risk of immunogenic reactions or transmission of infectious agents is negligible. The preparation and administration are straightforward, often performed in an outpatient setting, making it a viable option across a broad patient population [35]. Financially, PRP is relatively inexpensive, with single treatments costing between $300 and $700 depending on preparation protocols and clinical setting [30]. These costs are generally out-of-pocket expenses, as PRP is not consistently covered by insurance.

Conversely, BMAC involves additional procedural complexity and cost (Table 1). Harvesting BMAC requires an invasive aspiration procedure, most commonly from the iliac crest, which can result in donor site pain, hematoma, or, in rare cases, infection [36]. The need for sterile technique, surgical expertise, and specialized equipment makes BMAC significantly more expensive, ranging from $1500 to $2700 per treatment [36]. Despite these challenges, BMAC offers a cellular component that may provide sustained regenerative support, particularly in biologically compromised or revision ACL cases. While PRP and intraoperative BMAC are typically regulated under the U.S. Food and Drug Administration (FDA)’s 361 Human Cells, Tissues, and Cellular and Tissue-Based Products (HCT/P) designation as minimally manipulated autologous biologics, expanded or engineered MSCs fall under the stricter 351 pathway, which requires premarket approval through a Biologics License Application (BLA) due to their classification as more than minimally manipulated products [37].

A 2023 Markov decision analysis evaluated three strategies for isolated meniscal repair biologically analogous to ACL reconstruction, namely PRP augmentation, marrow venting procedure (MVP), and repair without biologic adjuncts. It found that both PRP and MVP yielded more quality-adjusted life years (QALYs) compared to standard repair. However, PRP came at a significantly higher cost, with an incremental cost-effectiveness ratio (ICER) of $161,742 per QALY, far above the standard $50,000 willingness-to-pay threshold. In contrast, the MVP approach produced similar QALY gains at a substantially lower cost, making it the more economically viable option [38].

Beyond direct costs, indirect factors such as faster return to work or reduced need for revision surgeries may further influence the economic appeal of biologic therapies. However, the literature remains limited in long-term cost–benefit analyses, particularly for BMAC [10]. Additionally, reimbursement policies vary widely, with most payers regarding these interventions as experimental. As biologic augmentation grows more common, health policies must adapt to address clinical evidence, real-world effectiveness, and equitable access. Ultimately, economic feasibility will determine how widely these therapies are adopted for routine ACLR.

### 3.5. Practical Implications and Rehabilitation Integration

Biologic augmentation techniques hold the potential to significantly influence rehabilitation timelines by enhancing graft healing and maturation. Improved MRI-defined graft signal intensity following biologic interventions, such as PRP or BMAC, has been correlated with tissue revascularization, collagen remodeling, and more organized ligamentization [39]. These biological improvements may enable clinicians to consider earlier initiation of progressive loading, weight-bearing protocols, and neuromuscular re-education. Such early mobilization strategies have been shown in general ACL rehabilitation literature to promote proprioceptive recovery and improve long-term function. Beyzadeoglu et al. (2020) conducted a cohort MRI study and found that applying PRF during ACL reconstruction was associated with improved graft maturation scores, suggesting a positive effect on early healing [17]. However, evidence directly linking biologically enhanced MRI outcomes to clinically validated return-to-sport (RTS) acceleration remains limited. Most studies to date use surrogate endpoints, such as graft signal intensity or patient-reported knee scores, rather than objective RTS metrics. Furthermore, differences in imaging protocols, interpretation criteria, and biologic product standardization complicate attempts to define universal RTS readiness thresholds based on imaging alone [39,40].

Despite these limitations, interest in biologic-enhanced rehabilitation continues to grow, particularly among high-performance athletes and motivated patients. These individuals are often eager to shorten recovery timeframes and reduce their risk of graft failure or contralateral injury, both of which are elevated during the vulnerable post-reconstruction period. Some centers have adopted integrated care models where MRI findings post-biologic augmentation are discussed collaboratively between radiologists, surgeons, sports physicians, and physical therapists to tailor rehabilitation progression [41]. Thus, a multidisciplinary team delivers care through a clinician-led, shared decision-making model. The medical lead (surgeon or sports physician) oversees surgical readiness and safety, while the rehabilitation lead (physical therapist) directs daily progress and testing. The extended team may include athletic training, strength coaching, and specialist support. Governance is clear: both leads must agree on advancement to high-level activities like running once the criteria are met. The return-to-sport decision is a consensus.

The integration of biologics into ACL reconstruction and rehabilitation introduces critical considerations around patient selection, response monitoring, and cost–benefit balance. Studies like Spierings et al. (2025) have demonstrated significant individual variability in graft-derived cytokine profiles, independent of age, suggesting the existence of biologic ‘responders’ who might benefit most from augmentation [42,43]. Similarly, reviews have highlighted how excessive inflammation and uneven tendon-to-bone healing can impair outcomes, reinforcing the rationale for stratifying patients based on inflammatory or regenerative markers to personalize therapy [5]. Additionally, as wearable technologies and digital health platforms advance, it may become possible to correlate biologic-enhanced healing markers with kinetic and kinematic movement patterns during rehabilitation [44]. Ultimately, a successful biologic-enhanced rehabilitation model will require multidisciplinary collaboration, real-time outcome feedback, and rigorous clinical validation to transition from a conceptual promise to the standard of care.

## 4. Conclusions

### Future Directions and Research Gaps

Recent preclinical investigations have revealed a wide array of innovative biologic strategies aimed at improving tendon-to-bone healing in ACL reconstruction. Among these, peptide-based approaches, gene therapies, and exosome-enriched biologics are gaining traction due to their scalability and translational potential [8,45]. For example, in a murine ACL reconstruction model, a self-assembling KI24RGDS peptide hydrogel significantly improved zonal tendon-to-bone attachment, fibrocartilage formation, and early mechanical fixation strength [46]. This hydrogel mimics the extracellular matrix and supports endogenous cell recruitment and matrix deposition. Preclinical studies suggest that tissue-engineered ACL grafts enhanced with MSC affinity components can significantly improve tendon-to-bone integration. Building on this concept, Tang et al. (2021) developed a biomimetic biphasic electrospun scaffold in a rat ACL model, which supported MSC-based fibroblast differentiation in vitro and promoted ligament regeneration and joint stability in vivo, highlighting its potential to enhance tendon-to-bone healing [47]. Similarly, nanofiber-reinforced 3D acellular tendon scaffolds incorporating MSC-binding peptide motifs demonstrated enhanced tendon–bone attachment and structural regeneration in large animal models, as shown by Yu et al. (2024) [48].

Along with these peptide-based advances, adipose-derived stem cells (ADSCs) have also emerged as a compelling biologic adjunct for ACL repair [49,50]. In a rabbit ACL reconstruction model, Matsumoto et al. (2021) demonstrated that ADSC sheet application nearly doubled early graft failure load (~17 N → ~37 N at 2 weeks and ~29 N → ~47 N at 4 weeks), enhanced collagen fiber diameter and organization, and limited bone tunnel widening, despite diminishing differences beyond 8 weeks, suggesting an early mechanobiologic advantage of this technique [50]. However, these were animal models. Future studies must evaluate safety, optimal dosing, delivery vehicles such as cell sheets, extracellular vesicles, or scaffolds, and comparative efficacy versus existing biologics in human trials [51].

Recent preclinical work has also evaluated a variety of cell-free peptide-based strategies that offer innovative alternatives to conventional cell-based therapies. Additionally, tissue-engineered grafts incorporating MSC-affinity peptides have shown improved integration and biomechanical performance in large animal studies. These developments suggest that short, synthetic peptides could offer scalable, off-the-shelf biologic options that eliminate the need for cell harvesting or specialized processing.

Beyond peptides, other innovative strategies under investigation include gene therapy vectors, exosome-based therapies, and smart biomaterials that release growth factors in a temporally controlled fashion. Gene editing tools, such as CRISPR-Cas9, are being explored to locally upregulate anabolic signaling pathways, potentially enhancing ligament regeneration at the molecular level [52]. Similarly, exosomes derived from MSCs and other progenitor cells are under investigation for their ability to deliver signaling molecules such as microRNAs, growth factors, and cytokines that enhance angiogenesis, regulate macrophage polarization, and remodel extracellular matrix at the tendon–bone interface [53]. Although these technologies remain largely experimental, they represent potential directions for biologic augmentation in ACL repair.

The future of ACL biologic augmentation will also benefit from advances in biomaterials science. For example, biodegradable scaffolds loaded with peptide sequences or growth factors are being designed to mimic the native enthesis and to guide zonal tissue regeneration. Hybrid constructs combining multiple strategies such as scaffold + PRP + exosome or scaffold + BMAC + peptide are also being tested to optimize the healing microenvironment [8,26]. These combinatory approaches may hold the key to overcoming the limitations of single-modality therapies. A randomized control, double blind study involving PRP + BMAC in ACL reconstruction by Lin et al. (2024) showed improved tendon-femoral bone interface and increased laxity compared to the control (nonaugmented) group [54].

Before these promising therapies can be widely adopted, key challenges must be addressed. Their efficacy is so far limited to animal studies, and moving to human clinical use means carefully assessing dosage, delivery techniques, and long-term biocompatibility. Ultimately, human trials for these peptide-based and hybrid solutions are needed to prove their safety and effectiveness. Furthermore, large-scale trials that directly compare PRP and BMAC using standardized methods are essential to determine which is more effective [22].

Moving forward, the integration of imaging biomarkers, functional outcome metrics, and personalized rehabilitation protocols will be vital for tailoring biologic treatments to individual patient profiles. Advances in MRI, such as UTE-T2* and T2* relaxation mapping and ultrasound elastography, now enable real-time, quantitative monitoring of graft maturation and biomechanical properties, potentially transforming how we assess biologic activity and guide safe return-to-sport timelines [55]. Combined with machine learning and wearable sensor data, these tools could facilitate precision medicine approaches in sports orthopedics [56]. Ultimately, the success of biologic ACL augmentation will depend on multidisciplinary collaboration, rigorous trial design, and the development of standardized clinical guidelines informed by both basic science and clinical evidence.

## Figures and Tables

**Table 1:** A summary comparing the composition, mechanism of action, key benefits, and inherent limitations of PRP and BMAC to inform decision-making.

Biological Augmentation	Platelet-Rich Plasma (PRP)	Bone Marrow Aspirate Concentrate (BMAC)
Core Composition	Concentrate of autologous platelets, growth factors, and fibrinogen.	Heterogeneous concentrate of autologous cells including mesenchymal stem cells (MSCs), hematopoietic stem cells, platelets, and growth factors.
Primary Mechanism of Action	Delivery of a supraphysiologic dose of growth factors (e.g., VEGF, TGF-β, PDGF) to stimulate and accelerate native healing processes like angiogenesis, cell proliferation, and collagen synthesis.	Provides a cellular scaffold with osteogenic and chondrogenic potential. Stem cells can differentiate into target tissues, modulate the immune response, and secrete paracrine factors to orchestrate regeneration.
Key Advantages	- Minimally invasive harvest (peripheral venipuncture). - Relatively low cost and quick preparation. - Excellent safety profile with minimal risk of immunogenic reaction. - Wide availability and extensive clinical experience. - Significant heterogeneity in preparation systems (leukocyte-rich vs. poor, single vs. double spin).	- Contains progenitor cells with true regenerative potential. - Higher concentration of MSCs and other nucleated cells. - Potentially more potent effect on bone tunnel healing and enthesis formation. - May have a stronger anti-inflammatory and immunomodulatory effect.
Key Limitations/Challenges	- Significant heterogeneity in preparation systems (leukocyte-rich vs. poor, single vs. double spin). - Growth factor concentration and release kinetics are short-lived. - Limited evidence for structural improvement in high-quality human trials.	- Invasive and painful harvest from the iliac crest. - Higher cost and more complex preparation requiring specialized kits. - Variable MSC yield depending on patient age, comorbidities, and technique.

## Data Availability

No new data were created or analyzed in this study.

## Abbreviations

The following abbreviations are used in this manuscript:

ACL	Anterior Cruciate Ligament
PRP	Platelet-Rich Plasma
PRF	Platelet-Rich Fibrin
BMAC	Bone Marrow Aspirate Concentrate
MSC	Mesenchymal Stem Cell
EVs	Extracellular Vesicles
ADSCs	Adipose-Derived Stem Cells
ADRCs	Adipose-Derived Regenerative Cells
MRI	Magnetic Resonance Imaging
US	Ultrasound
UTE-T2*	Ultrashort Echo Time T2-star (advanced MRI mapping sequence)
T2*	T2-star relaxation time (MRI parameter sensitive to tissue microstructure and field inhomogeneity)
CRISPR-Cas9	Clustered Regularly Interspaced Short Palindromic Repeats/CRISPR-associated protein 9 (gene editing tool)
RCT	Randomized Controlled Trial
IKDC	International Knee Documentation Committee score
KOOS	Knee Injury and Osteoarthritis Outcome Score
Lysholm	Lysholm Knee Scoring Scale
PROs	Patient Reported Outcomes
DBM	Demineralized Bone Matrix
LP-PRP	Leukocyte-Poor Platelet Rich Plasma
LR-PRP	Leukocyte-Rich Platelet Rich Plasma
BLA	Biologics License Application
FDA	U.S. Food and Drug Administration
HCT/P	Human Cells, Tissues, and Cellular and Tissue-Based Products
VEGF	Vascular Endothelial Growth Factor
PDGF	Platelet-Derived Growth Factor
TGF-β	Transforming Growth Factor Beta
IL-1β	Interleukin-1 Beta
TNF-α	Tumor Necrosis Factor Alpha
MMPs	Matrix Metalloproteinases
TIMP-1	Tissue Inhibitor of Metalloproteinase-1
